# Berry-Treacher Collins Syndrome With Congenital Bell’s Palsy and Unilateral Anotia: Tongue-Tie Release Under General Anesthesia

**DOI:** 10.7759/cureus.13250

**Published:** 2021-02-09

**Authors:** Umesh Kumar Singh, Vamsi Krishna Uppalapati, Himanshu Kumar, Roushan Patel, Abhijit Kumar

**Affiliations:** 1 Department of Anaesthesiology, Tata Main Hospital, Jamshedpur, IND; 2 Department of Otolaryngology, Tata Main Hospital, Jamshedpur, IND

**Keywords:** shared airway, general anesthesia, anotia, berry-treacher collins syndrome, congenital bell’s palsy, goldenhar syndrome, tongue – tie release, difficult airway

## Abstract

The inherited disease of unilateral anotia and ipsilateral Bell’s palsy is exceedingly uncommon, but it has a few other clinical manifestations. The prevalence of anotia in combination with congenital Bell’s palsy is well-known by Berry-Treacher Collins and Goldenhar syndrome. Despite the prevalence of anotia in combination with Bell’s palsy, there have been relatively very few case reports about the corresponding conditions in India. The aim of the paper is to discuss the anesthesia plan for a seven-year-old boy who underwent surgery for tongue-tie release.

## Introduction

Congenital Bell’s palsy is a neurological condition caused by an upper motor neuron disorder. It is usually believed to be either a congenital or inherited defect. About 23 cases per 100,000 births per year of Bell’s palsy and 0.76 cases per 10,000 births per year of unilateral anotia are reported globally [1,2]. Bell’s palsy of developmental origin is found in conjunction with other anomalies, including that of the pinna, external auditory canal, and laryngeal nerve [3]. Acquired causes of Bell’s palsy include perinatal trauma, intrauterine distress, and intrapartum insult. Anotia is a rare congenital malformation characterized by the complete absence of pinna, which may often include partial pinna closure or complete absence of the ear canal [4]. Pure tone audiometry can reveal unilateral or bilateral hearing loss and conductive hearing loss. Anotia is believed to be hereditary with no specific etiological factors [5].

The correlation between hearing loss and cranial nerve injury has been discovered to exist in children whose mothers were exposed to thalidomide during the antenatal period. Anotia syndrome has been linked with congenital Bell’s palsy and some cases display teratogenic symptoms. Well-known syndromes related to the subject include Berry-Treacher Collins and Goldenhar. A case report on the syndrome with complete unilateral anotia and ipsilateral Bell's palsy is presented [6]. Goldenhar syndrome shares the same Bell's characteristics with Treacher Collins syndrome, but is usually unilateral and asymmetrical, while Treacher Collins syndrome is usually bilateral and symmetrical. Individuals with Goldenhar syndrome tend to have vertebral defects and epibulbar dermoid more often [7]. Another significant anomaly related to Berry-Treacher Collins and Goldenhar is fissured tongue, which is also called ankyloglossia or tongue-tie; it is a congenital anomaly of the tongue [8]. We report an unusual case of a seven-year-old male who had anotia with ipsilateral Bell's palsy.

## Case presentation

**Figure 1:**
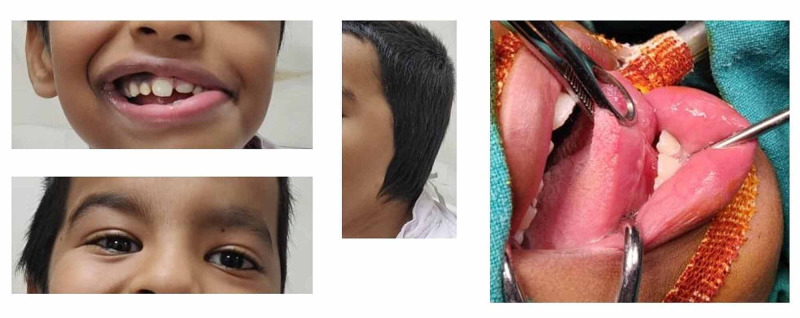
Physical characteristics of Bell's palsy

A seven-year-old boy presented with complaints of slurred speech, difficulty in breathing, and poor oral hygiene. The boy had a preexisting condition of congenitally ipsilateral Bell's palsy. His external physical appearance showed a complete absence of the left ear along with tongue-tie syndrome. Figure 1 illustrates the ophthalmological examination, which showed a regular multiple-tone palpebral fissure. The child had bilateral lower lid coloboma, meaning that he had minimum to no eyelashes at the lower lids.

Hearing assessment

On physical examination, the absence of the entire left ear and conductive hearing loss were identified, which require early intervention to promote natural language and auditory recovery.

Airway assessment

The patient had narrow airways and a history of snoring (choanal stenosis or atresia). The mouth opening was Mallampati III, which anticipated difficult airways.

Dental assessment

The dental assessment of the patient revealed slurred speech, dribbling of saliva, tongue-tie syndrome, and poor oral hygiene. 

**Figure 2:**
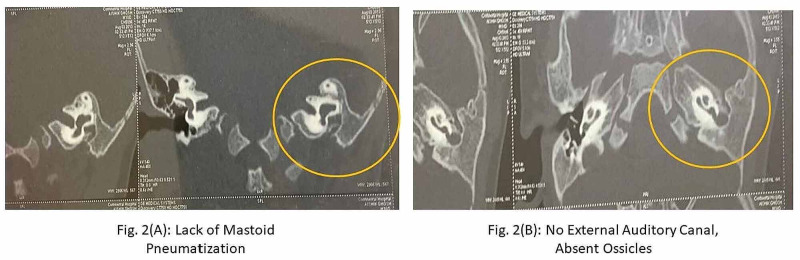
CT scan for identification of lack of pneumatized mastoid and missing left ear (A): the image shows a lack of pneumatized mastoid air cells, left lateral canal vestibular deformity (LCVD), and anomalous left facial never course. (B): the image shows a complete absence of the left ear, missing anterior and posterior mandibular bones on OPG, left EAC atresia (Altmann’s Group 3, Schuknecht Type D), hypoplastic left middle ear cavity with soft tissue and wall erosion, absent left middle ear ossicular system, and absent left cardio canal CT: computed tomography; OPG: orthopantomogram; EAC: external auditory canal

A CT scan of the patient was obtained (Figure 2).

A pediatric-age-group patient with Goldenhar syndrome along with narrow airway, undiagnosed cardiac anomalies, anticipated delayed recovery, and shared airway would be under very high risk when subjected to anesthesia. We decided to proceed with the surgery anyway.

## Discussion

Goldenhar/Treacher Collins syndrome has been identified in combination with congenital Bell’s palsy and anotia [9]. Apart from these conditions, intraoral anomalies like cleft lip or cleft palate are rare conditions with an incidence rate of one out of five million births. About 25% of the clinical presentations are associated with congenital heart and skeletal abnormalities [10,11].

Our patient was a seven-year-old boy with Berry-Treacher Collins and Goldenhar syndrome, and a history of snoring at night, who was admitted for tongue-tie release. His general physical examination revealed normal respiratory and cardiovascular parameters with no murmurs or palpable thrills. His CT scan showed that the bony and cartilaginous of the left external ear were atretic. The left middle ear ossicles were not seen. The middle ear cavity was hypoplastic and showed hypodense soft tissues in it, with the presence of bony erosion involving the floor and media wall of the left middle ear. There was a possible bony erosion and communication between the basal cochlear duct and the middle ear soft tissue near the expected location of the oval window. The oval and round windows were not well made. The mastoid air cell system was not pneumatized. The left facial nerve showed an abnormal course. The vestibular portion of the facial canal was normal. Further laterally, the facial canal was seen to descend anteriorly in the anterior wall of the middle ear cavity. The cochlea showed a normal number of turns. The posterior and superior semicircular canals were normal. The lateral semicircular canal and the vestibule were dilated and formed into a common chamber. The left carotid canal was absent. The patient underwent a gold standard approach for tongue-tie release on general anesthesia with endotracheal intubation and controlled mode ventilation.

The patient, who weighed 20 kg, was put under general anesthesia [12], primarily induced with the inhalational agent sevoflurane. After securing 20 gauze IV cannula, the maintenance of anesthesia was done with an injection of midazolam 0.5 mg; the opioid of choice was fentanyl 40 mcs; the anti-sylologue agent glycopyrrolate 150 mCg, the induction agent propofol 40 mg, and the muscle relaxant of choice atracurium 10 mg were also administered. After three minutes of the bag and mask ventilation, the airway was secured with a 3.5 cuffed Portex endotracheal tube through direct laryngoscopy technique (Cormack Lehane grade one); throat packing was done to prevent further aspiration; interop hemodynamics were stable. At the end of the surgery, the neuromuscular block was reversed adequately by administering neostigmine 1300 mCg and glycopyrrolate of 200 mCg and extubated after thorough suctioning; the throat packing was removed. The postoperative course was uneventful.

## Conclusions

We presented a unique case of a seven-year-old boy with Goldenhar syndrome who presented for tongue-tie release; based on our assessment, we anticipated difficult airway and shared airway for surgical access, and this made the case distinctive. With thorough preoperative evaluation and meticulous intraoperative measures, the surgery was successful without any mortality and morbidity.

## References

[1] Andrade EC, Júnior VS, Didoni AL, Freitas PZ, Carneiro AF, Yoshimoto FR (2005). Treacher Collins Syndrome with choanal atresia: a case report and review of disease features. Braz J Otorhinolaryngol.

[2] Harris J, Källén B, Robert E (1996). The epidemiology of anotia and microtia. J Med Genet.

[3] Bonar BE, Owens RW (1929). Bilateral congenital facial paralysis: review of the literature and a classification. Am J Dis Child.

[4] Gupta PV, Hegde AM (2016). Treacher Collins syndrome. Pediatric Dentistry for Special Child.

[5] Al Mosawi AJ (2019). The syndrome of congenital facial palsy and unilateral anotia. Clin Res Trials.

[6] Mahale RR, Mehta A, John AA, Buddaraju K, Shankar AK, Rangasetty S (2016). Newborn with congenital facial palsy and bilateral anotia/atresia of external auditory canal: rare occurrence. J Pediatr Neurosci.

[7] Sharma R, Narang P, Reddy YG, Sharma AK (2013). A triad of developmental anomalies-an unusual case. J Clin Diagn Res.

[8] Suryavanshi MP, Sodhi SJ, Kale LM, Rathod SJ, Kadam VD (2017). Berry syndrome: a case report and review of literature. J Indian Acad Oral Med Radiol.

[9] Girisha KM, Phadke SR (2005). Anotia and facial palsy: unusual features of cardiofacial syndrome. Indian J Pediatr.

[10] Tsitouridis I, Bintoudi A, Diamantopoulou A, Michaelides M (2007). Treacher-collins syndrome and associated abnormalities. A case report. Neuroradiol J.

[11] Chang CC, Steinbacher DM (2012). Treacher Collins syndrome. Semin Plast Surg.

[12] Spielberger L, Mazzia VD (1971). Anesthesia and Bell's palsy. Anesth Analg.

